# Preparation of Benzimidazole-Modified Resin and Its Adsorption Behavior Toward Cu(II) and Ni(II) Ions in Aqueous Media

**DOI:** 10.3390/ma19081532

**Published:** 2026-04-11

**Authors:** Keyu Chen, Yongming Wei, Kaihuai Duan

**Affiliations:** 1 School of Chemical Engineering, East China University of Science and Technology, Shanghai 200237, China; chenky@mail.ecust.edu.cn; 2 Membrane Science and Engineering R&D Lab, Shanghai 200237, China; 3 State Key Laboratory of Chemical Engineering, East China University of Science and Technology, Shanghai 200237, China; 13987981626@163.com

**Keywords:** 2-aminobenzimidazole, chelating resin, heavy metal adsorption

## Abstract

**Highlights:**

- A novel PS-2-AB chelating resin was synthesized by grafting 2-aminobenzimidazole onto polystyrene.
- The resin showed Cu(II) and Ni(II) adsorption capacities of 125.04 mg/g and 157.44 mg/g, respectively.
- The resin maintained over 84% adsorption capacity after five regeneration cycles.

**Abstract:**

To address heavy metal contamination in wastewater, this study developed a novel chelating resin (PS-2-AB) by grafting 2-aminobenzimidazole onto chloromethylated polystyrene. The resin was characterized using SEM, BET, FTIR, and XPS to confirm successful modification and analyze its structural properties. Batch adsorption tests were conducted to evaluate its removal performance for Cu(II) and Ni(II) ions. Under optimal conditions (pH 5.0–7.0, dosage: 1.0 g/L), PS-2-AB achieved maximum adsorption capacities of 125.04 mg/g for Cu(II) and 157.44 mg/g for Ni(II), which are significantly higher than those of the commercial resin D113 (44.68 mg/g for Cu(II) and 25.17 mg/g for Ni(II)) under the same conditions. Adsorption kinetics followed the pseudo-second-order model, indicating chemisorption-dominated behavior, while equilibrium data fit the Langmuir model, suggesting monolayer adsorption. Thermodynamic parameters confirmed a spontaneous and endothermic process. After five regeneration cycles, PS-2-AB retained approximately 87% (Cu) and 89% (Ni) of its original capacity, demonstrating good reusability. These results indicate that PS-2-AB exhibits markedly better adsorption performance than D113, making it a promising and cost-effective adsorbent for the efficient removal of Cu(II) and Ni(II) from aqueous media.

## 1. Introduction

In recent years, the acceleration of globalization coupled with the exponential growth of industrial activities has led to the increasingly critical issue of heavy metal contamination in wastewater. The pollution caused by heavy metal ions permeates various facets of production and daily life, impacting areas such as drinking water safety, chemical manufacturing, electroplating, textile printing and dyeing, and mineral smelting. Therefore, it is of great importance to remediate heavy metal ion pollution in the environment [1,2,3]. Heavy metals, defined as metallic elements possessing a density greater than 4.0 g/cm^3^ (e.g., Pb, Cd, Cr, Ni, and Cu), are characterized by high toxicity and recalcitrance to degradation [4]. Readily absorbed by lower organisms such as algae and plankton from surrounding water, heavy metals undergo biomagnification along the food chain, culminating in direct threats to human health, including severe outcomes such as cancer and developmental abnormalities [5]. As elemental contaminants, heavy metals are not subject to the biodegradation or chemical decomposition typical of organic pollutants. Upon entering environmental matrices, their transformation is limited to changes in oxidation state, adsorption, or precipitation, with total elemental mass conserved within the local environment. Therefore, it is necessary to develop technologies for heavy metal ion treatment.

Currently, the prevailing treatment technologies for heavy metal-laden wastewater encompass chemical precipitation, electrochemical methods, membrane separation, as well as physicochemical adsorption utilizing ion exchange resins and chelating resins. Moreover, biological methods such as phytoremediation and algal adsorption offer promising alternatives for heavy metal ion removal [6,7].

Chemical precipitation facilitates the removal of metal ions by introducing precipitants into wastewater, where the precipitants combine with the metal ions to form insoluble precipitates. Common precipitates include metal hydroxides, metal sulfides, and ferrites [8]. In a study by Benalia et al. [9], chemical precipitation using Ca(OH)_2_, NaOH, and Na_2_CO_3_ was employed to treat mixed heavy metals in industrial wastewater from the cable industry, achieving removal efficiencies exceeding 90%. This method is characterized by low cost and operational simplicity, making it one of the most widely adopted approaches for heavy metal treatment. However, its drawbacks include the substantial consumption of precipitants and the generation of sludge, which may pose risks of secondary environmental contamination.

Electrochemical techniques for heavy metal ion removal mainly comprise electrocoagulation and electrodeposition. In comparison to conventional precipitation methods, electrochemical approaches offer distinct advantages, including enhanced environmental compatibility, reduced reliance on chemical additives, and a lower propensity for secondary contamination. Al-Shannag et al. [10] employed an electrocoagulation reactor equipped with carbon steel electrodes to treat heavy metal ions in water, achieving removal efficiencies exceeding 97%, with complete removal (100%) observed for certain metal ions after one hour of operation. Guan et al. [11] developed a coupled electrodeposition and electrocatalysis process for nickel recovery from nickel-ammonia complex wastewater, attaining a recovery efficiency of 90%. Electrochemical methods exhibit high treatment efficacy and are capable of effectively removing heavy metals complexed with chelating agents, with minimal secondary pollution generation. However, their applications are subject to several limitations, including high energy consumption and the requirement that the wastewater to be treated possesses sufficient electrical conductivity [12].

Traditional methods for heavy metal ion treatment have inherent limitations and drawbacks. Chelating resins, as a class of emerging adsorbent materials, offer a new solution to this problem by virtue of their unique structural characteristics and adsorption properties [13,14].

Chelating resins are functional polymeric materials featuring specific active functional groups, including amino, carboxyl, thiol, and oxime moieties. The coordination atoms (e.g., N, O, S) present on the polymer backbone engage in coordination interactions with heavy metal ions, resulting in the formation of structurally stable chelate complexes [15]. These resins typically exhibit robust resistance to acids, alkalis, and corrosive environments, thereby maintaining satisfactory performance across a wide range of operating conditions. In comparison to traditional metal ion treatment technologies, chelating resins impose less stringent requirements on the application environment, obviate the need for complex equipment or procedures, and are amenable to facile elution using chemical reagents. They also demonstrate favorable environmental compatibility and regenerability, consistent with the principles of green chemistry [16,17].

Chelating resins featuring nitrogen atoms as coordination centers play a pivotal role in heavy metal ion adsorption research. Nitrogen atoms possess five outer-shell electrons, three of which participate in covalent bonding with other atoms, while the remaining two constitute a lone pair of electrons, endowing it with strong coordination capability and enabling the formation of stable coordination bonds with metal ions [18]. In chelating resins, nitrogen is commonly present in functional groups such as amino, pyridine, Schiff base, hydroxamic acid, and imidazole moieties.

Zou et al. synthesized a novel poly(chloromethylstyrene)-based chelating resin (CPS-TA) functionalized with a terpyridine-aniline side group [19]. Abundant nitrogen coordination sites are furnished by the three pyridine rings, resulting in adsorption capacities of 5.02, 3.38, and 1.27 mmol/g for Cu^2+^, Ni^2+^, and Pb^2+^, respectively. Bahsaine et al. [20] prepared a chelating resin via modification of Merrifield resin (MHL) using diethylenetriamine (DETA). The obtained resin showed 95.88% removal efficiency for Cr(III) and a maximum adsorption capacity of 38.35 mg/g.

Imidazole and its derivatives, as nitrogen-containing heterocyclic compounds, offer distinct advantages, including low synthesis costs and the ready availability of raw materials. In the pharmaceutical industry, imidazole-based compounds have been extensively utilized in drug design and development, demonstrating favorable biocompatibility and functional versatility [20]. However, among the existing chelating resins, reports on the application of imidazole-containing chelating resins for heavy metal adsorption are relatively scarce. Metal ions exhibit strong coordination affinity toward imidazole, accommodating various coordination numbers, with coordination typically occurring via the lone pair of electrons to form symmetric structures around the central metal ion, leading to stable metal-imidazole complexes [21]. Consequently, imidazole-based chelating resins hold considerable promise and research significance in the field of heavy metal wastewater treatment, warranting further systematic investigation and development.

In this study, a novel imidazole-based chelating resin adsorbent (PS-2-AB) was developed using 2-aminobenzimidazole (2-AB) and chloromethylated polystyrene resin (PS) as raw materials. The material is designed to remove heavy metal ions from aqueous solutions. It exhibits excellent coordination affinity toward metal ions, enabling efficient adsorption of Cu^2+^ and Ni^2+^ from water, along with good desorption performance and excellent reusability. The resin was systematically characterized, and the effects of key parameters such as adsorption temperature, solution pH, and adsorbent dosage on the adsorption capacity were investigated. Furthermore, the kinetics and thermodynamics of the adsorption process were studied to elucidate the underlying adsorption mechanism.

Compared with existing chelating resins, the innovations of this study are as follows:

(1) 2-Aminobenzimidazole was grafted onto chloromethylated polystyrene for the first time to prepare a novel imidazole-based chelating resin; the use of imidazole groups for heavy metal adsorption has been rarely reported.
(2) The adsorption capacities of PS-2-AB for Cu(II) and Ni(II) are significantly higher than those of the commercial resin D113 and rank at an above-average level among existing reports.
(3) After multiple cycles, the resin still maintains a high adsorption capacity, demonstrating good reusability and economic potential.

## 2. Materials and Methods

### 2.1. Materials

Chloromethylated polystyrene resin (PS, crosslinking degree: 2%, 40–50 mesh, analytical grade) was purchased from Shanghai Aladdin Biochemical Technology Co., Ltd. (Shanghai, China) D113 weak acid large pore cationic resin was purchased from Shanghai Titan Scientific Co., Ltd. (Shanghai, China) 2-aminobenzimidazole (2-AB, 99%) was purchased from Shanghai Adamas Reagent Co., Ltd. (Shanghai, China) N, N-dimethylformamide (DMF, 99.5%), ethanol (99.9%), acetone (99.9%), K_2_CO_3_ (99%), NiSO_4_·6H_2_O (99.9%), CuSO_4_·5H_2_O (99.5%), NaOH (99%), and hydrochloric acid (HCl, 38%) were purchased from Sinopharm (China) Chemical Reagent Co., Ltd. (Shanghai, China).

### 2.2. Synthesis of PS-2-AB

Chloromethylated polystyrene resin (PS) was sequentially washed with deionized water, acetone, and DMF (three times each) to remove impurities. After vacuum filtration, the resin was stored in a wide-mouth bottle.

**Figure 1:**
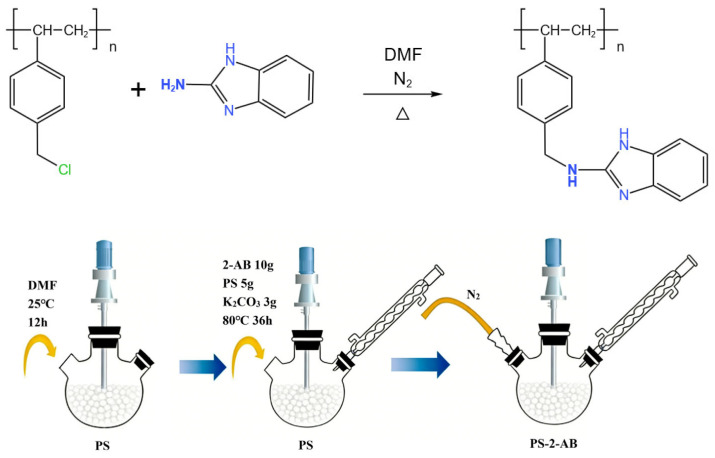
Reaction equation and preparation flowchart for PS-2-AB.

PS (5.00 g) was accurately weighed into a 100 mL three-necked flask, followed by addition of DMF (50 mL). The mixture was mechanically stirred at room temperature for 12 h to allow swelling. Following the swelling step, 2-aminobenzimidazole (10.00 g) and K_2_CO_3_ (3 g) were introduced into the three-necked flask. After ultrasonication to ensure complete dissolution, the reaction mixture was heated to 80 °C in a constant-temperature water bath under a nitrogen atmosphere, with the stirring speed maintained at 180 r/min. The reaction was carried out for 36 h under continuous reflux condensation. The synthetic route for the resin is illustrated in Figure 1. The reaction mechanism involves the nucleophilic substitution between the amino nitrogen atom of 2-aminobenzimidazole (2-AB) and the chlorine atom on the chloromethylated polystyrene resin (PS), resulting in the grafting of 2-AB onto the resin backbone. Based on this reaction mechanism, the synthesized resin is designated as PS-2-AB.

After reaction, the mixture was cooled and filtered. The resin was washed successively with DMF, ethanol, and dilute HCl, and then rinsed with deionized water with shaking until it reached neutral pH. The purified resin was vacuum-filtered for 2 min and dried in a vacuum oven to constant weight, yielding approximately 8.2 g of off-yellow resin for subsequent characterization and adsorption experiments. The graft yield (GD) was calculated to be approximately 64% based on the mass difference of the resin before and after grafting.

$$\mathrm{GD}=\frac{\left(W_{a}-W_{b}\right)}{W_{b}}\times 100\%$$

*W_a_*: Mass of resin after grafting reaction and thorough drying (g);

*W_b_*: Mass of chloromethylated polystyrene resin before grafting (g).

### 2.3. Scanning Electron Microscopy (SEM)

The as-synthesized chelating resin was subjected to vacuum drying, followed by dispersion in ethanol and sputter-coating with a thin layer of gold for sample preparation. Surface morphology analysis was performed using a ZEISS Sigma 300 scanning electron microscope.

### 2.4. Brunauer–Emmett–Teller (BET)

The resin was characterized by nitrogen adsorption–desorption measurements. The analysis was performed in mesopore mode, with a degassing time of 6 h prior to measurement. The instrument model is the Micromeritics ASAP 2460.

### 2.5. Fourier Transform Infrared Spectroscopy (FT-IR)

The as-synthesized resin sample was vacuum dried and subsequently ground into a fine powder. FTIR spectra were collected in transmission mode, via the KBr pellet method across the 400–4000 cm^−1^ range, using a Thermo Fisher Scientific Nicolet iS20 spectrometer.

### 2.6. X-Ray Photoelectron Spectroscopy (XPS)

XPS was primarily employed to investigate the chemical states of elements within the chelating resin both prior to and following the adsorption of heavy metal ions. The acquired spectral data were subsequently subjected to peak fitting using CasaXPS (version 2.3) software to determine the elemental composition and binding energies of the samples. The instrument model is the Thermo Scientific K-Alpha.

### 2.7. Ion Affinity Experiment

To clarify the affinity order of the resin for target metal ions and evaluate its adsorption selectivity in the presence of coexisting interfering ions, a cation affinity experiment was designed. The experimental procedure is as follows: A specific amount of resin was weighed and placed into a 200 mL conical flask to which, 50 mL of mixed solution containing Cu^2+^ and Ni^2+^, along with Zn^2+^, Mg^2+^, Ca^2+^, Na^+^, and other coexisting ions at equal initial concentrations, was added. The conical flask was then positioned in an isothermal water bath oscillator and oscillated at 150 rpm until adsorption equilibrium was reached. Subsequently, the concentration of each heavy metal ion before and after adsorption was determined using ICP-MS, from which, the equilibrium adsorbed amount qₑ was calculated according to Equation (1). By comparing the adsorbed amounts, the metal ion with the highest affinity for the resin was identified, providing a basis for subsequent studies on adsorption selectivity.

(1) $$Q_{e}=\frac{\left(C_{0}-C_{e}\right)V}{m}$$

Q_e_: equilibrium adsorption capacity of the chelating resin for a given metal ion (mg/g).

C_0_: initial concentration of the heavy metal ion in the aqueous phase (mg/L).

C_e_: equilibrium concentration of the heavy metal ion in the liquid phase (mg/L).

V: volume of the metal ion solution employed (L).

m: mass of the resin used in the experiment (g).

### 2.8. Static Adsorption Experiment

Static adsorption tests were performed under controlled conditions (temperature, concentration, time) to evaluate the resin’s adsorption capacity for heavy metal ions. The resin was added separately to Ni(II) and Cu(II) solutions, followed by shaking in a constant-temperature water bath shaker at 150 rpm for about 8 h until equilibrium. The metal ion concentrations before and after adsorption were measured by ICP-MS, and the equilibrium adsorption capacity was calculated using Equation (1). Meanwhile, the adsorption performance of PS-2-AB resin for heavy metal ions was evaluated by comparing it with that of the commercial weak acid cation adsorption resin D113.

### 2.9. Competitive Adsorption Experiment

This experiment investigated the competitive adsorption behavior between nickel ions and copper ions in solution. Specifically, a binary system containing equal concentrations of Cu^2+^ and Ni^2+^ was prepared, to which an aliquot of resin was added and the mixture was oscillated at 150 rpm until equilibrium was reached using an isothermal water bath oscillator. Subsequently, the adsorbed amounts qₑ were quantified by measuring the changes in heavy metal ion concentrations via ICP-MS before and after the process according to Equation (1). This provides insight into how each ion competes for binding sites on the resin surface.

### 2.10. Static Desorption Experiment

Static desorption experiments were conducted to assess the reusability and economic feasibility of the chelating resin. The metal-loaded resin was first rinsed with ultrapure water, followed by desorption using 0.10 mol/L HCl aqueous solution as the eluent under constant-temperature shaking. After desorption, the resin was sequentially washed with acetone and ultrapure water, dried, and subsequently reused in the next adsorption cycle to evaluate changes in adsorption performance. The desorption efficiency was calculated using Equation (2), and the regeneration performance of the chelating resin in static adsorption was evaluated by comparing the adsorption capacities obtained from successive cycles.

(2) $$\;\;\eta =\frac{m_{e}}{m_{0}}\times 100\%$$

η: desorption efficiency of the chelating resin;

m_0_: previously measured maximum adsorption capacity per unit mass of the resin (mg/g);

m_e_: mass of heavy metal ions eluted per unit mass of the resin (mg/g).

### 2.11. Kinetic Adsorption Experiment

Adsorption kinetics experiments are essential for elucidating the underlying mechanism of the adsorption process. The kinetic data obtained from these experiments were fitted to kinetic models to determine the type of adsorption process and evaluate the rate-limiting factors. A typical experiment began with transferring a 20 mL aliquot of heavy metal ion solution of predetermined concentration into a 100 mL conical flask, after which, the chelating resin was added. The resulting mixture was subsequently shaken at 150 rpm in a thermostatic water bath shaker. At various time intervals, 0.5 mL aliquots were sampled for subsequent analysis. Kinetic data for resin adsorption were collected from experiments at various temperatures.

The kinetic data were analyzed by fitting to the pseudo-first-order and pseudo-second-order kinetic models [22]. The corresponding model equations are given as follows:

Pseudo-first-order kinetic model:

(3) $$q_{t}=q_{e}(1-e^{-k_{1}t})$$

q_t_: adsorption capacity at time t (mg/g);

q_e_: equilibrium adsorption capacity (mg/g);

k_1_: pseudo-first-order rate constant (min^−1^);

t: adsorption time (min).

Linear fitting of the pseudo-first-order kinetic model was performed by plotting $ln\;(q_{e}-q_{t})$ against *t*.

Pseudo-second-order kinetic model:

(4) $$\frac{t}{q_{t}}=\frac{1}{k_{2}{q_{e}}^{2}}+\frac{t}{q_{e}}$$

k_2_: pseudo-second-order rate constant (g·mg^−1^·min^−1^).

Linear fitting of the pseudo-second-order kinetic model was performed by plotting $t/q_{t}$ against *t*.

### 2.12. Thermodynamic Adsorption Experiment

Adsorption thermodynamics experiments are essential for elucidating the underlying adsorption mechanism. The resulting thermodynamic data were fitted to thermodynamic models to investigate the relationship between the resin’s equilibrium adsorption capacity and the equilibrium concentration of the adsorbate.

In a typical procedure, a 20 mL aliquot of heavy metal ion solution at a predetermined concentration was transferred into a 100 mL conical flask, followed by the addition of the chelating resin. The mixture was then agitated in a thermostatic water bath shaker at 150 rpm. After adsorption equilibrium was attained, samples were collected for analysis. Experiments were conducted at different temperatures to obtain comprehensive thermodynamic data for the adsorption process of the resin.

The obtained thermodynamic data were subsequently analyzed by fitting to the Langmuir and Freundlich isotherm models. The Langmuir model is one of the most widely employed theoretical models in adsorption thermodynamics. However, its applicability is not universal, as it is based on certain assumptions [23].

If the adsorption process of heavy metal ions onto the chelating resin can be described by the Langmuir model, it follows the linear equation below:

(5) $$\frac{C_{e}}{Q_{e}}=\frac{1}{k_{L}Q_{max}}+\frac{C_{e}}{Q_{max}}$$

Q_max_: maximum Langmuir adsorption capacity (mg/g);

Q_e_: equilibrium adsorption capacity (mg/g);

*C_e_*: equilibrium metal ion concentration (mg/L);

*K_L_*: Langmuir constant (L/mg).

Linear fitting of the Langmuir model was performed by plotting *C_e_*/*Q_e_* against *C_e_*. When the temperature increases, if *K_L_* increases accordingly, it indicates that the reaction is endothermic; conversely, if *K_L_* decreases, the reaction is exothermic.

The Freundlich model is primarily employed to describe multilayer adsorption occurring on heterogeneous solid surfaces with non-uniform active sites [24]. If the adsorption process of heavy metal ions onto the chelating resin can be described by the Freundlich model, it follows the linear equation:

(6) $$\;lnQ_{e}=lnK_{F}+\frac{1}{n}lnC_{e}$$

*K_F_*: Freundlich constant.

$\frac{1}{n}$ serves as a key indicator of the nonlinearity of the adsorption process and the surface heterogeneity, thereby also reflecting the favorability of adsorption. Linear fitting of the Freundlich model was performed by plotting $\ln Q_{e}$ versus $\ln C_{e}$. A value of 1/*n* within the range of 0 to 1 indicates favorable adsorption, with a smaller 1/*n* value corresponding to greater surface heterogeneity [25].

### 2.13. Thermodynamic Parameter Calculation

Thermodynamic parameters were calculated to characterize the adsorption behavior, including its spontaneity and driving forces, as well as the changes in system disorder and thermal effects. The parameter expressions are as follows:

(7) $$K_{D}=\frac{q_{e}}{C_{e}}$$

(8) $$lnK_{D}=\frac{∆S}{R}-\frac{∆H}{RT}$$

(9) ∆G=∆H−∆ST

R: 8.314 J·mol^−1^·K^−1^;

*T*: adsorption temperature (K);

*K_D_*: standard equilibrium constant;

∆H: enthalpy change of the adsorption process (kJ/mol);

∆G: Gibbs free energy change (kJ/mol).

Linear fitting of the data was performed by plotting $\ln K_{D}$ against $T^{-1}$, from which, the thermodynamic parameters ΔS, ΔH, and ΔG were calculated.

## 3. Results

### 3.1. SEM

**Figure 2:**
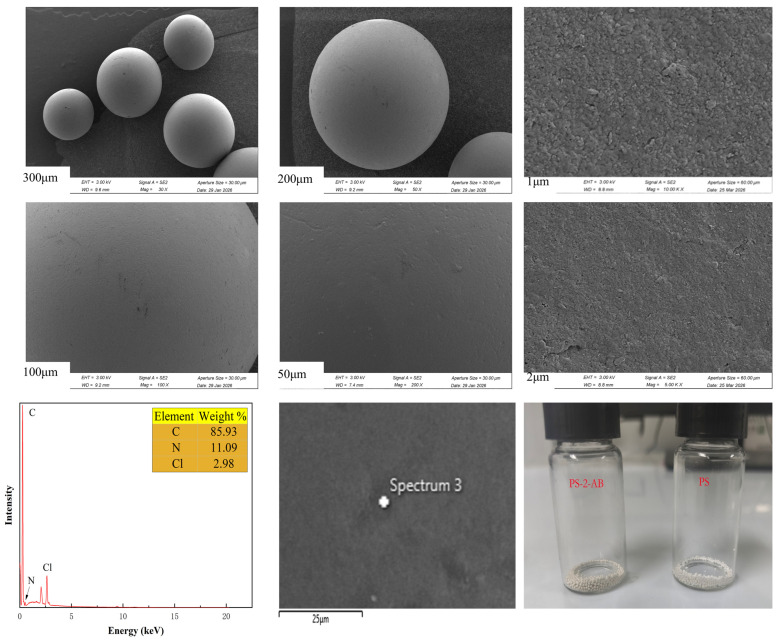
SEM Image, EDS Spectrum, and Photograph of PS-2-AB Resin.

The SEM and EDS characterization results, and the actual picture of PS-2-AB are shown in Figure 2. In low-magnification images, all visible particles exhibit near-spherical morphology with no apparent fractures, irregular shapes, or agglomerations, indicating excellent molding control during resin synthesis. Minor light scratches and tiny point impurities were observed but no structural defects were found, suggesting good mechanical strength and chemical stability of the resin. At high magnifications, the surface displays a coralline rough structure accompanied by numerous pores, forming a multi-level composite architecture that significantly increases the specific surface area and provides abundant active sites for adsorption, thereby endowing the resin with superior adsorption properties. EDS analysis confirmed the incorporation of nitrogen atoms while chlorine content decreased, indicating successful grafting of benzimidazole groups onto the resin backbone.

### 3.2. BET

**Figure 3:**
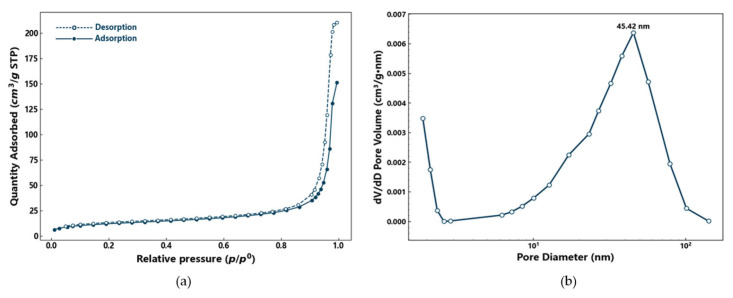
(**a**) Nitrogen adsorption–desorption isotherms of PS-2-AB resin; (**b**) Pore size distribution derived from the desorption branch.

The nitrogen adsorption–desorption isotherms of PS-2-AB resin (Figure 3a) are typical of type IV, which is characteristic of mesoporous materials. In the low-pressure region (p/p_0_ < 0.4), a slow, slightly convex increase in adsorption indicates weak nitrogen–surface interactions and negligible monolayer adsorption. Multilayer adsorption gradually occurs as pressure increases. A pronounced hysteresis loop appears at p/p_0_ > 0.6, with the desorption branch above the adsorption branch, reflecting complex pore structures. The absence of a saturation plateau in the latter half of the isotherm suggests a wide mesopore size distribution, with capillary condensation spanning a broad pressure range. Overall, PS-2-AB resin is predominantly mesoporous with few micropores, and adsorption is governed by pore filling. The pore size distribution (Figure 3b) confirms the highest proportion of mesopores, followed by macropores, and a lower content of micropores. The BET specific surface areas of PS and PS-2-AB are 36.2919 m^2^/g and 44.0722 m^2^/g, respectively, and their average pore diameters are 26.2364 nm and 29.5477 nm, respectively. The detailed data of pore volume and pore area are presented in Table 1.

### 3.3. FT-IR

**Figure 4:**
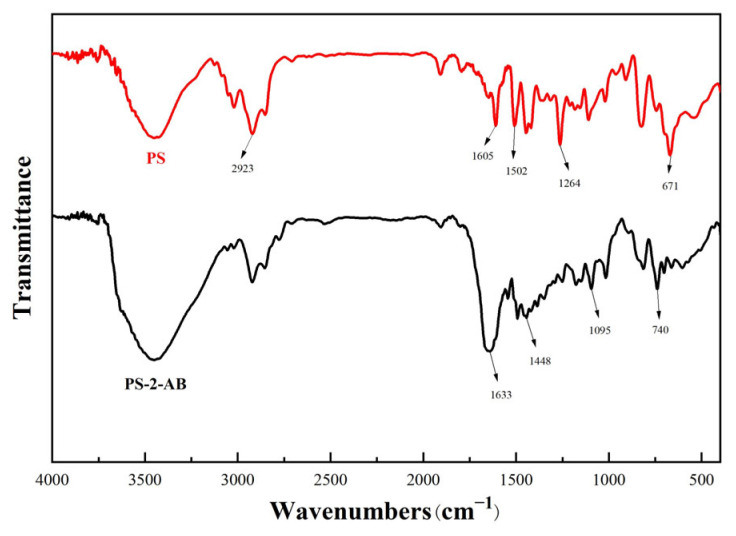
FTIR Spectra of PS-2-AB Resin Before and After Reaction.

Figure 4 shows the FTIR spectra of PS and PS-2-AB resin. The band at 2926 cm^−1^ is assigned to the asymmetric stretching of aliphatic –CH_2_– [26]. The bands at 1605, 1502, and 1448 cm^−1^ correspond to benzene ring C=C stretching, and the band at 1264 cm^−1^ arises from –CH bending on substituted benzene rings [27]. The characteristic –Cl stretching peak at 670 cm^−1^ confirms the starting material as chloromethylated polystyrene [28]. After grafting, new peaks emerge at 1095 cm^−1^ (C–N stretching of aliphatic amines) and 740 cm^−1^ (out-of-plane bending of benzimidazole ring H) [29]. The disappearance of the characteristic –CH_2_– (1264 cm^−1^) and –Cl (670 cm^−1^) peaks of the chloromethylated resin indicates successful grafting of benzimidazole groups onto the resin matrix.

### 3.4. XPS

**Figure 5:**
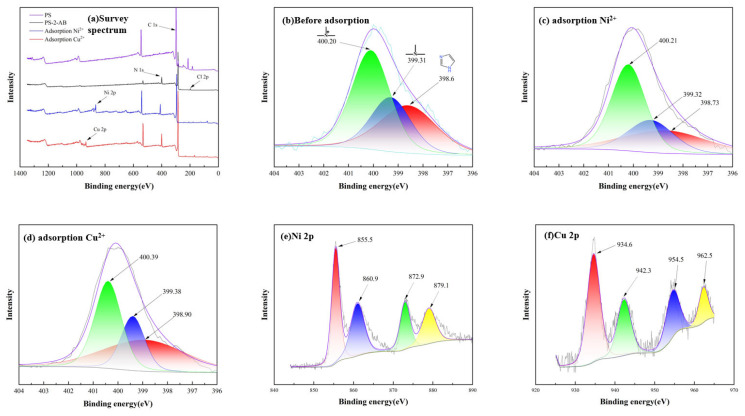
XPS Spectra of PS-2-AB Resin: (**a**) Survey Spectrum; (**b**) N1s Spectrum Before Adsorption; (**c**) N1s Spectrum After Ni^2+^ Adsorption; (**d**) N1s Spectrum After Cu^2+^ Adsorption; (**e**) Ni2p Spectrum; (**f**) Cu2p Spectrum.

As shown in Figure 5a, the XPS survey spectra of the resin before/after grafting and after Cu^2+^/Ni^2+^ adsorption reveal the emergence of a N peak and alterations in the C peak after grafting, confirming successful grafting. The N 1s spectrum of the resin before adsorption is presented in Figure 5b. For the pristine resin, the fitted N1s peaks are as follows: Peak 1 (N1) at 398.6 eV corresponds to the characteristic peak of the N atoms (–N=) in the imidazole ring; Peak 2 (N2) at 399.3 eV is attributed to the amine groups introduced by 8-aminobenzimidazole; and Peak 3 (N3) at 400.2 eV is the characteristic peak of protonated N atoms (–N^+^=) in the imidazole ring [30,31].

The N1s spectrum after Ni^2+^ adsorption is shown in Figure 5c. Following Ni^2+^ adsorption, the binding energy of the –N= peak shifts to 398.73 eV, which is attributed to the coordination between the nitrogen atoms in the imidazole ring and Ni^2+^.

The N1s spectrum after Cu^2+^ adsorption is shown in Figure 5d. After adsorption, the binding energy of the –N= peak increases to 398.90 eV, which is consistent with the findings of Wang et al., who reported an increase in the binding energy of the coordinating nitrogen atoms in imidazole-containing Cu^2+^ complexes after reaction [32].

Figure 5e,f show the Ni2p and Cu2p spectra of the resin after heavy metal ion adsorption, respectively. For the Ni2p spectrum, the peaks at 855.5 eV and 860.9 eV correspond to the Ni2p_3_/_2_ main peak and satellite peak, respectively, while those at 872.9 eV and 879.1 eV are assigned to the Ni2p_1_/_2_ main peak and satellite peak [33]. For the Cu2p spectrum, the peaks at 934.6 eV and 954.5 eV are the two spin-orbit splitting peaks of Cu2p, while the peaks at 942.3 eV and 962.5 eV are the characteristic shake-up satellite peaks of Cu^2+^ [34].

### 3.5. Adsorption Mechanisms

**Figure 6:**
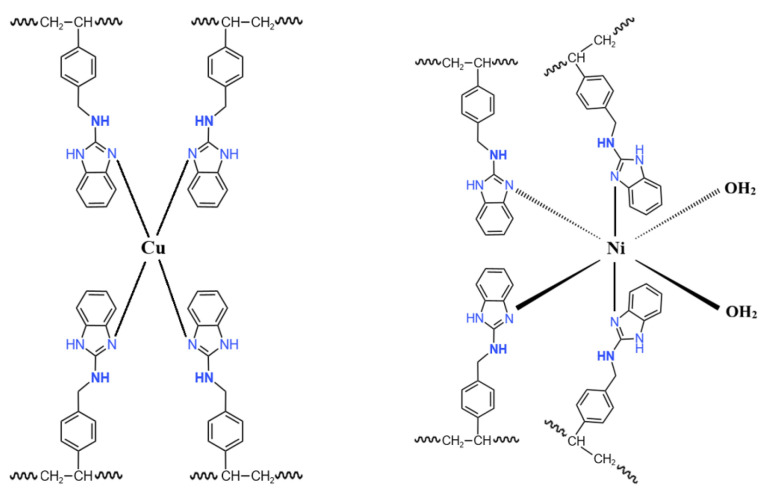
Schematic Diagram of Possible Coordination Structures Between PS-2-AB Resin and Cu^2+^/Ni^2+^.

Combined FTIR and XPS analyses reveal that the coordination mode of PS-2-AB resin with Cu^2+^ and Ni^2+^ is monodentate, involving exclusively the imine nitrogen (–N=) of the imidazole ring, while the amino nitrogen (–NH–) does not participate. The XPS N1s spectra show that upon adsorption of Ni^2+^ or Cu^2+^, only the –N= peak (398.6 eV) shifts to 398.73 eV and 398.90 eV, respectively, whereas the amino nitrogen peak (399.3 eV) remains essentially unchanged, confirming the absence of electron cloud density transfer. In the FTIR spectra, the characteristic peaks of the polystyrene main chain (2926, 1605, 1502, 1448 cm^−1^) remain unaltered before and after adsorption, and the peaks at 1095 cm^−1^ (C–N stretching) and 740 cm^−1^ (out-of-plane bending of benzimidazole) persist, indicating that metal–N bonds are formed exclusively on the pendant benzimidazole groups without modifying the main chain structure. The central Cu^2+^ relies on four imine nitrogen atoms (–N=) from four distinct benzimidazole rings to complete its coordination. These four nitrogen atoms originate from four different side chains on the resin matrix, spatially surrounding Cu^2+^ to form a crosslinked coordination center. This structure is reasonable in solid-phase adsorption: under high grafting density, Cu^2+^ can simultaneously coordinate with four adjacent imine nitrogen atoms, analogous to the “Cu^2+^ coordinated with four benzimidazole nitrogen atoms” structure reported in the literature [35]. Similarly, Ni(II) also relies on four imine nitrogen atoms for coordination. However, due to the absence of the Jahn–Teller effect, its geometry is a distorted octahedron (coordination number 6), with the four imine nitrogen atoms occupying the equatorial plane and two axial sites coordinated by water molecules [36]. Figure 6 illustrates the proposed coordination structures formed between Cu^2+^/Ni^2+^ and the resin.

### 3.6. Ion Affinity Experiment

**Figure 7:**
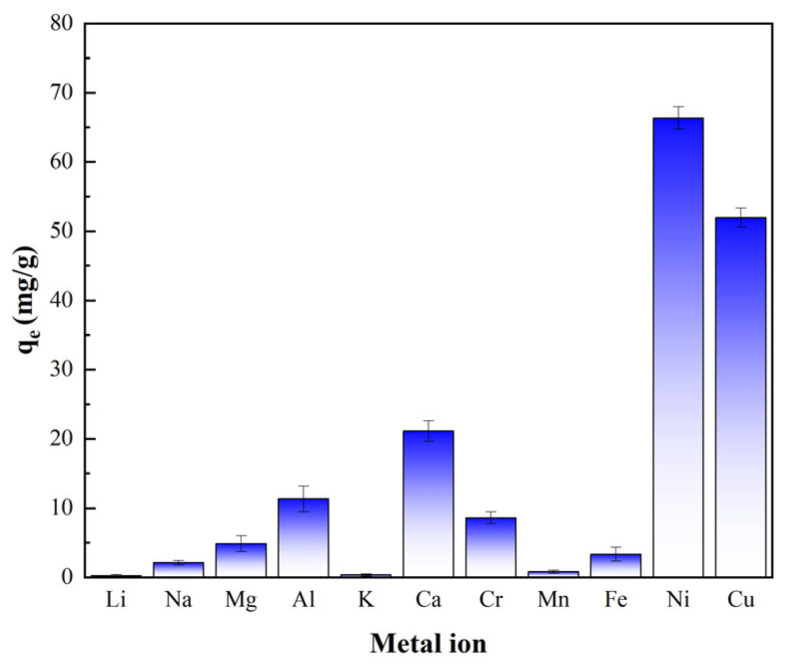
Adsorption Capacity of PS-2-AB Resin for Various Metal Ions.

A 40 mL mixed solution containing multiple metal ions, each at an initial concentration of 100 mg/L, was prepared. The pH was adjusted to 5.0 (in this study, 0.1 mol/L HCl and NaOH aqueous solutions were used). PS-2-AB resin (0.02 g) was added, and the mixture was oscillated until adsorption equilibrium is reached; then, the equilibrium adsorbed amount for each ion was calculated. All charted data in this paper that includes error bars are based on triplicate experiments. The results of the ion affinity experiment are shown in Figure 7.

As shown in Figure 7, the adsorption performance of PS-2-AB resin for different metal ions exhibits significant differences. The equilibrium adsorption capacities for ions such as Li^+^, Na^+^, K^+^, and Mn^2+^ are extremely low, indicating almost no adsorption. For ions such as Mg^2+^, Al^3+^, Cr^3+^, Ca^2+^, and Fe^3+^, the adsorption capacities are relatively low. However, the resin maintains a relatively high adsorption capacity for Ni^2+^ and Cu^2+^. This is because the ionic radii and electronic structures of Cu^2+^ and Ni^2+^ are suitable, allowing them to easily form thermodynamically stable five-membered or six-membered chelate rings with the ligand. In contrast, chelates formed by other transition metals may have lower stability due to greater ring tension or insufficient bond energy. These results indicate that the resin possesses strong anti-interference ability in multi-ion coexistence systems and can effectively distinguish and selectively adsorb Ni^2+^ and Cu^2+^.

### 3.7. Static Adsorption Experiment

#### 3.7.1. Effect of Resin Dosage Experiment

**Figure 8:**
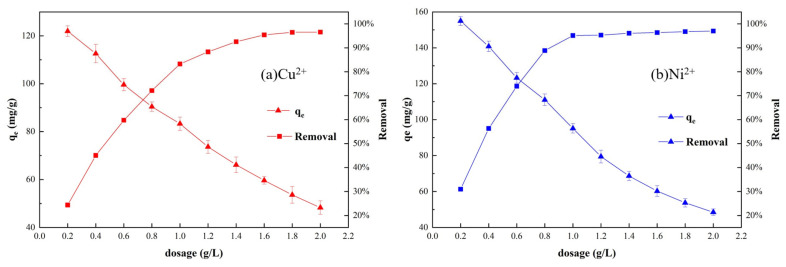
Effect of PS-2-AB Resin Dosage on Static Adsorption: (**a**) Effect on Adsorption Capacity and Removal Rate of Cu^2+^; (**b**) Effect on Adsorption Capacity and Removal Rate of Ni^2+^.

The effects of PS-2-AB resin dosage on the adsorption capacity and ion removal efficiency were investigated over a range of 0.2 g/L to 2.0 g/L, and the results are shown in Figure 8.

The removal efficiency of metal ions increases with increasing resin dosage. When the resin dosage reaches a certain level, the removal efficiency remains essentially unchanged, indicating that the resin approaches saturated adsorption. This is because at low resin dosages, the concentration of metal ions in solution is high while the available adsorption sites are insufficient, resulting in low removal efficiency. As the resin dosage increases, the number of adsorption sites increases, leading to improved removal efficiency. When the dosage increases to a critical point, the removal efficiency gradually approaches 100% and enters a plateau region. This is attributed to the fixed total amount of metal ions in solution; excessive adsorption sites compete for limited ions, causing unsaturated adsorption at each site and consequently reducing the utilization efficiency of the resin. To facilitate subsequent experiments, a resin dosage of 1.0 g/L was selected for both ions, at which, the removal efficiencies of Ni^2+^ and Cu^2+^ were 95.13% and 83.26%, respectively.

#### 3.7.2. Effect of pH Experiment

**Figure 9:**
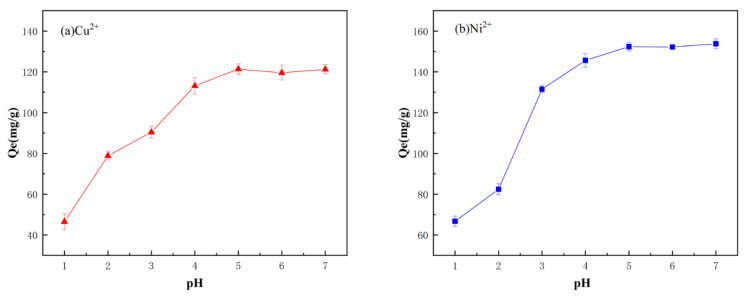
Effect of Solution pH on Static Adsorption of PS-2-AB Resin: (**a**) Effect on Adsorption Capacity of Cu^2+^; (**b**) Effect on Adsorption Capacity of Ni^2+^.

The pH of the metal ion solutions was adjusted within the range of 1.0–7.0 by adding 0.1 mol/L HCl and NaOH. The pH dependence of adsorption is depicted in Figure 9, where Figure 9a shows that the adsorption capacity of the resin for Cu^2+^ was relatively low at pHs below 4.0. This is attributed to the presence of a large amount of H^+^ from hydrochloric acid in the solution, leading to competitive adsorption between H^+^ and Cu^2+^ for the limited adsorption sites on the resin. Additionally, the imidazole groups on the PS-2-AB resin are readily protonated, which may also contribute to the decrease in Cu^2+^ adsorption capacity. As the pH increased, the adsorption capacity for Cu^2+^ also increased, reaching a maximum at approximately pH 5.0. When the pH exceeded 5.6, copper hydroxide precipitation occurred. As shown in Figure 9b, the adsorption capacity for Ni^2+^ reached a maximum at around pH 5.0, and nickel hydroxide precipitation was observed when the pH reached approximately 7.7. In summary, for subsequent adsorption experiments, the pH of the Cu^2+^ solution was adjusted to 5.0 and that of the Ni^2+^ solution to 7.0 to achieve maximum adsorption capacities.

#### 3.7.3. Effect of Temperature Experiment

**Figure 10:**
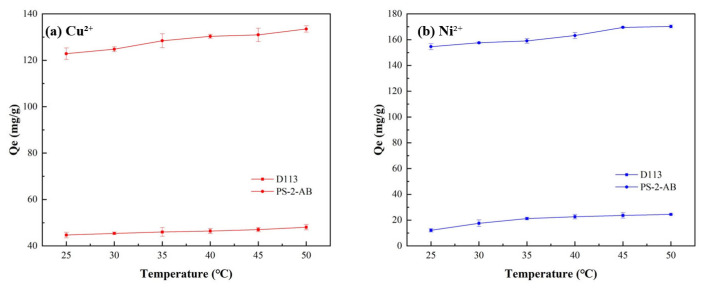
Effect of Adsorption Temperature on Static Adsorption of PS-2-AB and D113 Resins: (**a**) Effect on Adsorption Capacity of Cu^2+^; (**b**) Effect on Adsorption Capacity of Ni^2+^.

Adsorption experiments were conducted at 25–50 °C in 5 °C increments; the results are presented in Figure 10. Increasing the temperature was found to be favorable for the adsorption process of both PS-2-AB and D113 resins. This is likely because the adsorption involves the formation of chemical bonds (i.e., coordination bonds between the chelating resin and metal ions), which requires a certain activation energy. Elevated temperatures can provide the necessary energy to overcome this energy barrier.

#### 3.7.4. Effect of Time Experiment

**Figure 11:**
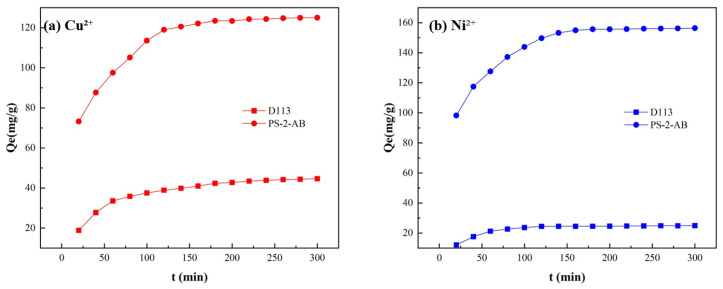
Effect of Adsorption Time on Static Adsorption of PS-2-AB and D113 Resins: (**a**) Effect on Adsorption Capacity of Cu^2+^; (**b**) Effect on Adsorption Capacity of Ni^2+^.

At 25 °C, 40 mL of a metal ion solution with a concentration of 200 mg/L was prepared, and samples were taken for measurement every 20 min. The results are shown in Figure 11. As illustrated in Figure 11a, the adsorption rate of Cu^2+^ onto PS-2-AB resin exhibited a trend of rapid increase followed by a slower increase, with the growth rate of adsorption gradually decreasing, and saturation was essentially reached at approximately 180 min. As shown in Figure 11b, the adsorption of Ni^2+^ onto PS-2-AB resin reached saturation at approximately 140 min, and the saturated adsorption capacity was higher than that of Cu^2+^, indicating that the resin exhibits better static adsorption performance for Ni^2+^ under the same conditions. For D113 resin, the adsorption of Cu^2+^ reached saturation at approximately 150 min, and the adsorption of Ni^2+^ reached saturation at approximately 100 min, with adsorption capacities lower than those of PS-2-AB resin.

#### 3.7.5. Effect of Initial Concentration Experiment

**Figure 12:**
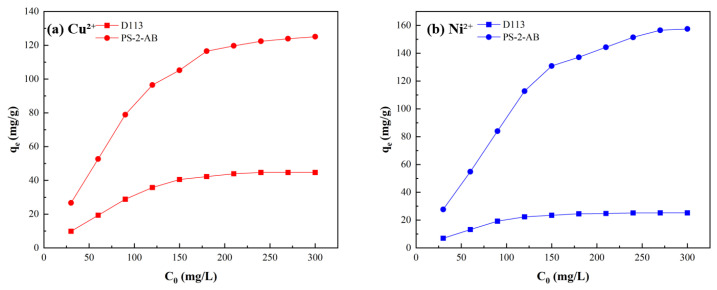
Effect of Solution Concentration on Static Adsorption of PS-2-AB and D113 Resins: (**a**) Effect on Adsorption Capacity of Cu^2+^; (**b**) Effect on Adsorption Capacity of Ni^2+^.

At 25 °C, metal ion solutions were prepared at concentrations of 30–300 mg/L, and the results are shown in Figure 12. At low initial metal ion concentrations, the adsorption capacity of PS-2-AB resin for heavy metal ions increased rapidly. When the concentration reached approximately 180 mg/L, the static adsorption of Cu^2+^ onto the resin became essentially saturated, with further increases in concentration resulting in only a slight increase in adsorption capacity. At a concentration of 300 mg/L, the adsorption capacity reached a maximum of 125.04 mg/g. For Ni^2+^, the static adsorption became essentially saturated when the concentration reached approximately 240 mg/L, and the adsorption capacity increased only marginally with further increases in concentration, reaching a maximum of 157.44 mg/g at 300 mg/L. This behavior can be attributed to the fact that the initial concentration serves as the primary driving force for adsorption, significantly enhancing the adsorption capacity at lower concentrations. However, at higher concentrations, the adsorption sites on the resin become saturated, leading to a diminished increase in adsorption capacity. In comparison, the maximum adsorption capacities of D113 resin for Cu^2+^ and Ni^2+^ are 44.68 mg/g and 25.17 mg/g, respectively, both significantly lower than those of PS-2-AB resin.

### 3.8. Static Desorption Experiment

**Figure 13:**
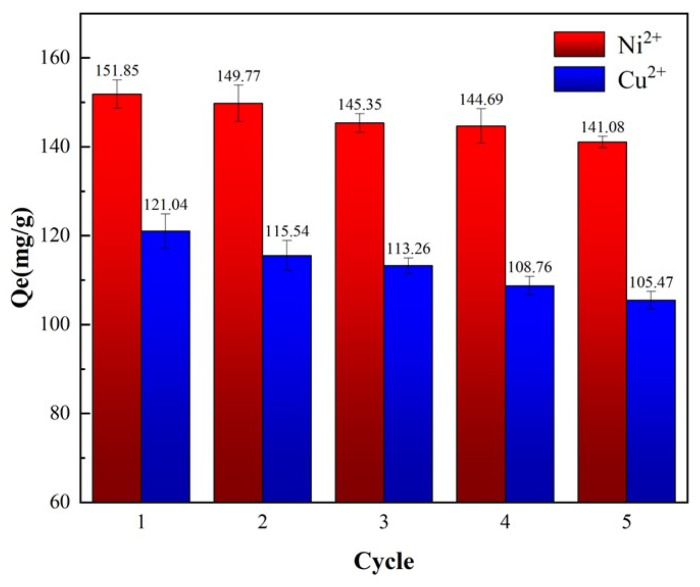
Static adsorption–desorption cyclic adsorption capacity of PS-2-AB resin.

A 500 mL stock solution of Cu^2+^ (400 mg/L) was prepared. For each adsorption cycle, 20 mL of this solution was mixed with PS-2-AB resin and shaken at 25 °C for 8 h. The resin was then eluted by shaking with 0.10 mol/L HCl for 6 h. This adsorption–desorption cycle was repeated five times. The same procedure was followed for Ni^2+^. Figure 13 shows the cyclic experiment results.

Following five cycles of adsorption and desorption, the adsorption capacity of the PS-2-AB chelating resin for Cu^2+^ remained at approximately 84% of its maximum adsorption capacity, while that for Ni^2+^ remained at approximately 89% of its maximum adsorption capacity. Meanwhile, the desorption rates were calculated according to Equations (2) and (3). The maximum adsorption capacities for both ions were determined in Section 3.7.5, and the results are presented in Table 2.

### 3.9. Competitive Adsorption Experiment

**Figure 14:**
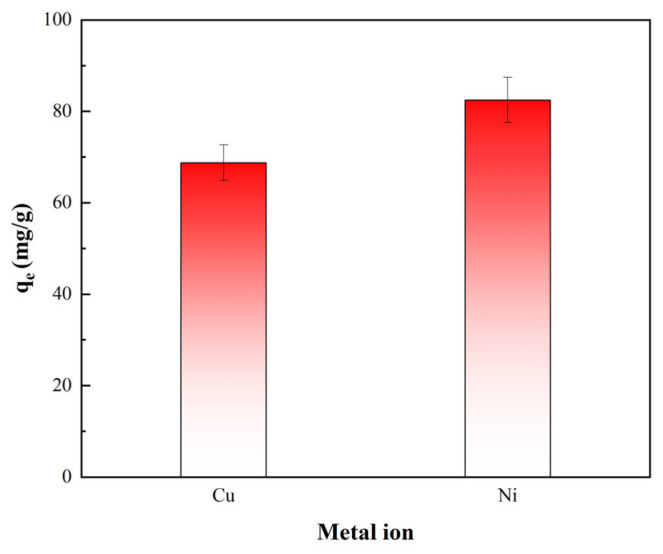
Competitive Adsorption of Cu^2+^ and Ni^2+^ onto PS-2-AB Resin.

A 40 mL volume of a binary mixed solution of Cu^2+^ and Ni^2+^ was prepared, with a pH of 5.0 and a concentration of 200 mg/L for each ion. Then, 0.02 g of PS-2-AB resin was added, and the mixture was oscillated until adsorption equilibrium was reached. The equilibrium adsorption capacities of the two ions were calculated. The results are shown in Figure 14.

Figure 14 shows that in the binary system containing both Ni^2+^ and Cu^2+^, the adsorption capacity of PS-2-AB resin for Ni^2+^ is higher than that for Cu^2+^. Compared with the single-component systems, the equilibrium adsorption capacities for both ions decreased significantly: from 157.44 mg/g to 82.49 mg/g for Ni^2+^, and from 125.05 mg/g to 68.76 mg/g for Cu^2+^, indicating a pronounced competitive adsorption between Ni^2+^ and Cu^2+^ as both ions compete for the active adsorption sites on the resin surface. Although the adsorption capacities of both ions declined, Ni^2+^ maintained a relatively higher adsorption level in the competitive system, suggesting that its affinity for the resin is superior to that of Cu^2+^, allowing it to preferentially occupy the active sites. Nevertheless, the resin still retains a considerable adsorption capacity for Cu^2+^ under competitive conditions, demonstrating that PS-2-AB remains effective for Cu^2+^ removal in multicomponent systems and exhibits promising potential for the treatment of wastewater containing multiple heavy metal ions.

### 3.10. Kinetic Adsorption Experiment

**Figure 15:**
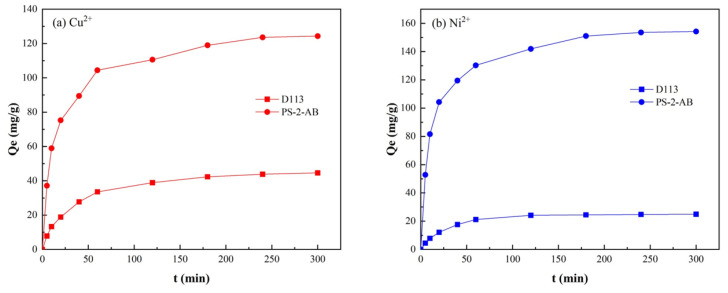
Adsorption Kinetics Curves for Adsorption of Cu^2+^ and Ni^2+^ onto PS-2-AB and D113 Resins: (**a**) Adsorption Kinetics Curve for Cu^2+^; (**b**) Adsorption Kinetics Curve for Ni^2+^.

Samples were obtained at periodic intervals to derive the adsorption kinetic profiles of PS-2-AB resin and D113 resin (Figure 15). As illustrated in Figure 15a, the static adsorption capacity of PS-2-AB resin for Cu^2+^ surged within the first 60 min and attained equilibrium at approximately 150 min, while that of D113 resin for Cu^2+^ reached equilibrium at approximately 170 min. Figure 15b shows that the adsorption capacity of PS-2-AB resin for Ni^2+^ climbed rapidly within the first 80 min and leveled off at around 120 min, whereas the adsorption capacity of D113 resin for Ni^2+^ reached equilibrium at approximately 100 min.

The curves were linearly fitted using the pseudo-first-order and pseudo-second-order kinetic models. The fitting results are shown in Figure 15, and the corresponding kinetic parameters are listed in Table 3.

**Figure 16:**
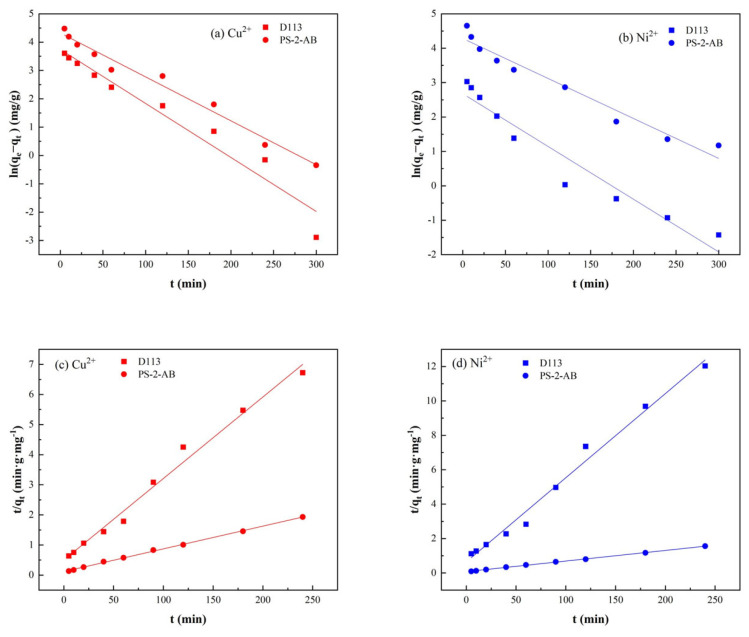
Linear Fitting of Adsorption Kinetic Models for Adsorption of Cu^2+^ and Ni^2+^ onto PS-2-AB and D113 Resins: (**a**) Pseudo-First-Order Kinetic Model for Cu^2+^; (**b**) Pseudo-First-Order Kinetic Model for Ni^2+^; (**c**) Pseudo-Second-Order Kinetic Model for Cu^2+^; (**d**) Pseudo-Second-Order Kinetic Model for Ni^2+^.

As shown in Figure 16, The pseudo-first-order model showed a poor fit, with linear regression coefficients ($R^{2}$) below 0.98. Conversely, the pseudo-second-order model provided a better fit for the adsorption onto both PS-2-AB and D113 resins, with R^2^ values exceeding 0.99, indicating a good fit. Moreover, the calculated equilibrium adsorption capacities ($q_{e}^{\mathrm{cal}}$) from the pseudo-second-order model for PS-2-AB resin were 129.870 mg/g for Cu^2+^ and 162.337 mg/g for Ni^2+^, respectively, in close agreement with the experimental values ($q_{e}^{\exp}$). For D113 resin, the calculated $q_{e}^{\mathrm{cal}}$ values were 39.639 mg/g for Cu^2+^ and 20.466 mg/g for Ni^2+^. This implies that the adsorption of heavy metal ions by the resins is controlled by chemical adsorption. In other words, the enrichment of heavy metal ions relies mainly on electron exchange and transfer between the surface imidazole groups of PS-2-AB resin and the metal ions, which form stable chelates.

### 3.11. Thermodynamic Adsorption Experiment

**Figure 17:**
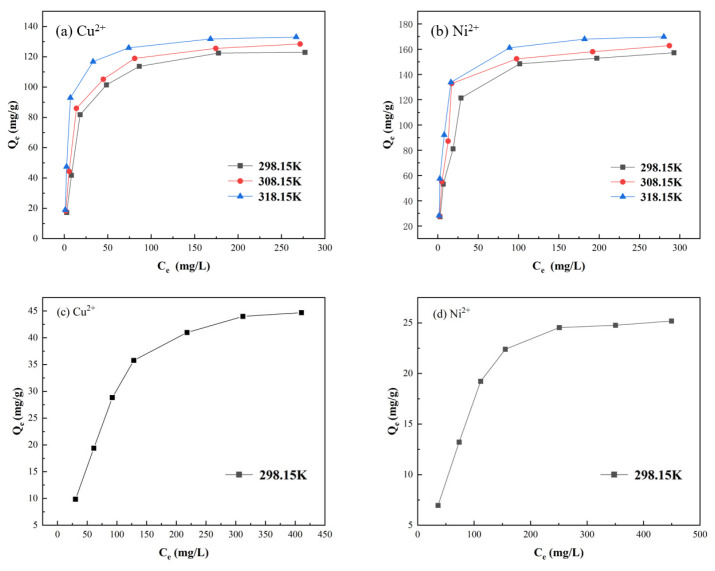
Adsorption Isotherms for Adsorption of Cu^2+^ and Ni^2+^ onto PS-2-AB and D113 Resins: (**a**) Cu^2+^ on PS-2-AB; (**b**) Ni^2+^ on PS-2-AB; (**c**) Cu^2+^ on D113; (**d**) Ni^2^ on D113.

The adsorption isotherms of PS-2-AB resin for Cu^2+^ and Ni^2+^ were measured at 25 °C, 35 °C, and 45 °C, with adsorption time intervals of 5, 10, 20, 40, 60, 90, 120, 180, and 240 min. The results are shown in Figure 17.

**Figure 18:**
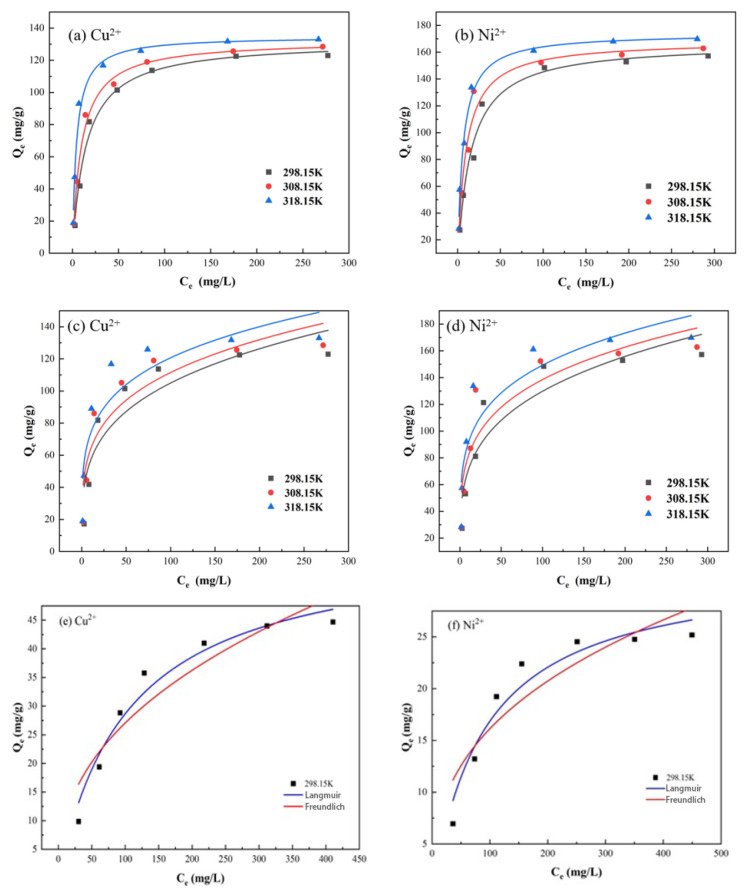
Langmuir and Freundlich Adsorption Isotherm Fitting Curves for Adsorption of Cu^2+^ and Ni^2+^ onto PS-2-AB and D113 Resins: (**a**) Langmuir Fitting for Cu^2+^ Adsorption onto PS-2-AB; (**b**) Langmuir Fitting for Ni^2+^ Adsorption onto PS-2-AB; (**c**) Freundlich Fitting for Cu^2+^ Adsorption onto PS-2-AB; (**d**) Freundlich Fitting for Ni^2+^ Adsorption onto PS-2-AB; (**e**) Both Langmuir and Freundlich Fittings for Cu^2+^ Adsorption onto D113; (**f**) Both Langmuir and Freundlich Fittings for Ni^2+^ Adsorption onto D113.

The experimental data were analyzed via the Langmuir and Freundlich isotherm models. The fitting results are shown in Figure 18, with the corresponding parameters listed in Table 4 and Table 5.

The fitting results in Figure 18 show that the Langmuir model describes the adsorption of Cu^2+^ and Ni^2+^ onto both PS-2-AB and D113 resins well, suggesting a monolayer adsorption mechanism. That is, adsorption occurs on uniformly distributed active sites with similar affinities for the metal ions. Furthermore, the maximum adsorption capacities ($q_{m}$) for Cu^2+^ and Ni^2+^ calculated using the Langmuir isotherm model at 25 °C for PS-2-AB resin are 131.69 mg/g and 166.87 mg/g, respectively, values that correspond closely to the previously measured data, indicating that PS-2-AB chelating resin possesses excellent adsorption performance. In contrast, the $q_{m}$ values for D113 resin under the same conditions are 58.96 mg/g for Cu^2+^ and 31.93 mg/g for Ni^2+^, both lower than those of PS-2-AB resin.

### 3.12. Thermodynamic Parameter Calculation

**Figure 19:**
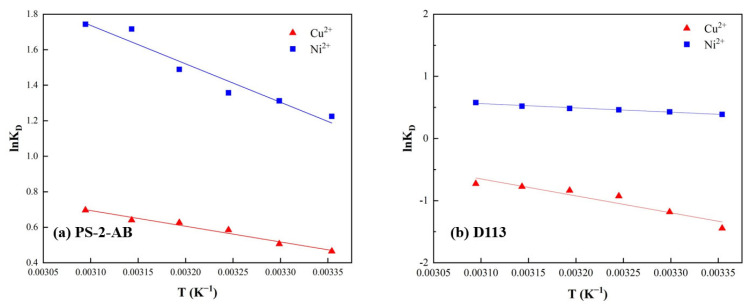
Linear Fitting of Thermodynamic Parameters for Adsorption onto PS-2-AB and D113 Resins: (**a**) PS-2-AB Resin; (**b**) D113 Resin.

The thermodynamic experimental data were linearly fitted (Figure 19), and the calculated parameters from the slopes and intercepts are listed in Table 6. To ensure that adsorption equilibrium was reached, an adsorption time of 6 h was used for all experiments. As the temperature increased, the adsorption equilibrium constants (K_D) for both ions increased, indicating an endothermic adsorption process. In addition, the calculated Gibbs free energy changes (ΔG) were all negative, thereby substantiating the spontaneity and endothermicity of the adsorption process.

It can be observed that as the temperature increases, the adsorption equilibrium constants ($K_{D}$) for both ions also increase, indicating that the adsorption process of PS-2-AB and D113 resins for the two ions is endothermic. Additionally, the calculated Gibbs free energy changes (ΔG) are all negative, further confirming that the adsorption process is spontaneous and endothermic.

## 4. Conclusions

In this study, a novel imidazole-based chelating resin, PS-2-AB, was successfully prepared using polystyrene resin (PS) as the matrix, along with 2-aminobenzimidazole (2-AB), N,N-dimethylformamide, and other reagents. The morphology, structure, and physical properties of the resin were characterized by SEM, FTIR, XPS, and BET. Static adsorption experiments for Cu^2+^ and Ni^2+^ were conducted to investigate the effects of temperature, pH, initial concentration, and contact time on adsorption performance. Furthermore, the adsorption mechanism, kinetics, and thermodynamics were systematically studied. The main conclusions are as follows:

(1) Various characterization techniques confirmed the successful modification of the PS-2-AB resin. XPS analysis revealed a significant shift in the binding energy of characteristic functional groups after modification, while FTIR further verified the immobilization of imidazole groups. BET and SEM analyses indicated that the resin possesses a favorable specific surface area and morphology, providing a structural basis for its excellent adsorption performance.
(2) Static adsorption experiments showed that the optimal resin dosage was 1.0 g/L. A moderate increase in pH facilitated adsorption, with maximum adsorption occurring at pH 5.0 for Cu^2+^ and pH 7.0 for Ni^2+^. Adsorption was enhanced by increasing temperature, initial metal ion concentration, and contact time. At 25 °C and an initial concentration of 300 mg/L, the maximum adsorption capacities of PS-2-AB for Cu^2+^ and Ni^2+^ were 125.04 mg/g and 157.44 mg/g, respectively. In contrast, the commercial D113 resin achieved only 44.68 mg/g for Cu^2+^ and 25.17 mg/g for Ni^2+^ under identical conditions, indicating that PS-2-AB possesses substantially superior adsorption performance.
(3) The adsorption kinetics were best fitted by the pseudo-second-order model, indicating chemical adsorption as the rate-controlling step. The adsorption thermodynamics were well described by the Langmuir isotherm model, suggesting a monolayer adsorption mechanism. Thermodynamic parameters showed that ΔG < 0, indicating that the adsorption process was spontaneous and endothermic.
(4) After five consecutive desorption–adsorption cycles, the adsorption capacities of the resin for Cu^2+^ and Ni^2+^ remained at approximately 87% and 89% of their maximum capacities, respectively, with high desorption rates and large adsorption capacities. This study demonstrates that the adsorption performance of PS-2-AB is not only significantly superior to that of D113, but also ranks at an above-average level among the existing resins listed in Appendix A. Therefore, it represents an efficient, low-cost, and highly promising adsorbent material for the effective removal of Cu(II) and Ni(II) from aqueous solutions.

## Figures and Tables

**Table 1:** Pore distribution of PS-2-AB resin and PS.

Resin	Pore Size Range	Pore Volume		Pore Area	
		cm^3^/g	Percent	m^2^/g	Percent
PS-2-AB	0.35–2 nm	0.004	1.29%	10.957	25.00%
	2–50 nm	0.163	50.34%	23.100	52.71%
	50–300 nm	0.156	48.37%	9.768	22.29%
PS	0.35–2 nm	0.0015	0.72%	3.163	9.99%
	2–50 nm	0.119	57.25%	24.120	76.19%
	50–300 nm	0.0873	42.03%	4.372	13.82%

**Table 2:** Desorption rate of PS-2-AB resin.

Ion	Cycle 2	Cycle 3	Cycle 4	Cycle 5	Cycle 6
Ni^2+^	95.18%	91.90%	91.01%	89.42%	87.94%
Cu^2+^	92.27%	90.11%	87.56%	83.88%	80.69%

**Table 3:** Linear Fitting Parameters of Adsorption Kinetics for Cu^2+^ and Ni^2+^ onto PS-2-AB and D113 Resins.

Resin	Ion	${q_{e}}^{exp}$ (mg∙g^−1^)	Pseudo-First-Order Kinetic Model			Pseudo-Second-Order Kinetic Model		
			${q_{e}}^{cal}$ (mg∙g^−1^)	$k_{1}$ (min^−1^)	$R^{2}$	${q_{e}}^{cal}$ (mg∙g^−1^)	*k_2_* (g∙mg^−1^∙min^−1^)	$R^{2}$
PS-2-AB	Cu^2+^	125.04	82.127	0.02074	0.9862	129.870	0.00051	0.9955
	Ni^2+^	157.44	75.446	0.01528	0.9319	162.337	0.00064	0.9989
D113	Cu^2+^	44.68	42.216	0.01096	0.9441	39.639	0.00015	0.9859
	Ni^2+^	25.17	72.513	0.01162	0.9521	20.466	0.00037	0.9857

**Table 4:** Langmuir Isotherm Model Fitting Parameters for Adsorption of Cu^2+^ and Ni^2+^ onto PS-2-AB and D113 Resins.

Model	Resin	Ion	T (K)	$K_{L}$	$q_{m}$ (mg/g)	$R^{2}$
Langmuir	PS-2-AB	Cu^2+^	298.15	0.0694	131.69	0.9854
			308.15	0.1045	132.46	0.9898
			318.15	0.2312	134.92	0.9831
		Ni^2+^	298.15	0.0673	166.87	0.9818
			308.15	0.1062	168.60	0.9746
			318.15	0.1619	174.15	0.9880
	D113	Cu^2+^	298.15	0.0095	58.96	0.9662
		Ni^2+^	298.15	0.0112	31.93	0.9425

**Table 5:** Freundlich Isotherm Model Fitting Parameters for Adsorption of Cu^2+^ and Ni^2+^ onto PS-2-AB and D113 Resins.

Model	Resin	Ion	T (K)	$K_{F}$	*n*	$R^{2}$
Freundlich	PS-2-AB	Cu^2+^	298.15	30.64	3.74	0.8251
			308.15	36.45	4.12	0.8399
			318.15	48.25	4.98	0.8493
		Ni^2+^	298.15	38.42	3.78	0.8548
			308.15	47.28	4.29	0.8113
			318.15	55.28	4.64	0.8251
	D113	Cu^2+^	298.15	3.88	2.37	0.8870
		Ni^2+^	298.15	3.05	2.76	0.8331

**Table 6:** Fitted parameters of the Freundlich isotherm model for the adsorption of Cu^2+^ and Ni^2+^ onto PS-2-AB and D113 resin.

Resin	Ion	∆G (kJ∙mol^−1^)						∆H (kJ∙mol^−1^)	∆S (J∙mol^−1^∙K^−1^)	$R^{2}$
		298.15 K	303.15 K	308.15 K	313.15 K	318.15 K	323.15 K			
PS-2-AB	Cu^2+^	−1.164	−1.306	−1.449	−1.592	−1.734	−1.877	7.345	28.538	0.9669
	Ni^2+^	−2.943	−3.294	−3.645	−3.996	−4.347	−4.698	17.988	70.202	0.9296
D113	Cu^2+^	−3.671	−3.995	−4.318	−4.643	−4.966	−5.290	15.642	64.776	0.9793
	Ni^2+^	−0.955	−1.067	−1.179	−1.291	−1.403	−1.515	5.721	22.393	0.8776

## Data Availability

The original contributions presented in this study are included in the article. Further inquiries can be directed to the corresponding author.

## Abbreviations

The following abbreviations are used in this manuscript:

SEM	Scanning electron microscopy
BET	Brunauer–Emmett–Teller
FT-IR	Fourier transform infrared spectroscopy
XPS	X-ray photoelectron spectroscopy
PS	Chloromethylated polystyrene resin
PS-2-AB	Polystyrene resin grafted with 2-aminobenzimidazole
DMF	N,N-Dimethylformamide

## Appendix A

**Table A1:** Comparison of adsorption performance between PS-2-AB and existing resin adsorbents.

Resin	Functional Group	Matrix	Cu(II) Adsorption Capacity (mg/g)	Ni(II) Adsorption Capacity (mg/g)
PS-2-AB	2-Aminobenzimidazole	Chloromethylated polystyrene	125.04	157.44
PVI-g-PS	1-Vinylimidazole	Chloromethylated polystyrene	52.7	103.9
CPS-AA	Schiff base	Chloromethylated polystyrene	237.7	168.5
D113	carboxyl	Macroporous acrylic	44.68	25.17
001×7	Sulfonic acid group	Macroporous styrene-divinylbenzene	40–50	25–35
Dowex M-4195	bis-picolylamine	Macroporous styrene-divinylbenzene	86.44	300
